# Use of Extended Cover Factor Theory in UV Protection of Woven Fabric

**DOI:** 10.3390/polym13081188

**Published:** 2021-04-07

**Authors:** Klara Kostajnšek, Krste Dimitrovski

**Affiliations:** Department of Textiles, Graphics Art and Design, Faculty for Natural Sciences and Engineering, University of Ljubljana, Snežniška 5, 1000 Ljubljana, Slovenia; krste.dimitrovski@gmail.com

**Keywords:** cover factor, simplified model of UV penetration, woven fabrics simulation, coefficient K_R_, K_T_ and K_A_ determination

## Abstract

The paper presents an extension of existed cover factor theory more suitable for the evaluation of light penetration through a net woven fabrics structure. It also introduces a new simplified model of predicting the ultraviolet (UV) protective properties of woven fabrics assuming that the coefficient of reflection (K_R_), transmission (K_T_), and absorption (K_A_) of constitutive yarns are known. Since usually they are not, the procedure of preparation of simulation of proper woven fabric samples without interlacing and with known constructional parameters is also presented. The procedure finishes with a fast and cheap detection of missed coefficient for any type of yarns. There are differences between theoretical and measured results, which are not particularly significant in regard to the purpose and demands of investigation.

## 1. Introduction

In recent decades, protection against harmful ultraviolet (UV) radiation has become increasingly important, mainly due to the depletion of the ozone layer in the atmosphere, but also due to exposure to artificial sources. Preventive measures to protect the skin and eyes are now common knowledge. These include limiting exposure to the sun daily, using sunscreens and sunglasses, and wearing protective clothing and headgear at the same time. Of all these measures, the use of mechanical means of protection, which physically prevent the negative effects of UV rays, helps to protect the skin most effectively. Most of the protection of the skin from ultraviolet radiation is represented by clothing made of woven or knitted fabrics of suitable construction. The purpose of clothing is to protect us from external atmospheric and climatic factors while providing comfort [1]. The advantage of clothing products compared to chemo pharmaceuticals is that textiles do not need to be applied directly to and/or on the skin, avoiding the occurrence of many allergies and other chemical reactions with the skin [2] and preventing a negative impact on the environment [3]. At the same time, the advantage is that we can easily separate the covered protected body surface from the unprotected during application.

The protection of textile products and their quality is determined by many parameters, such as type of fibers and raw material composition [2,4,5,6]. Better protection is generally provided by synthetic fibers (e.g., polyester (PET)), due to their molecular structure, while natural ones (e.g., cotton (CO)), except for wool, offer poorer protection [2,7]. The second important parameter is material construction [8,9,10], where the high cover factor or closeness of the fabric structure, depending on the type of weave and the diameter of the yarn, prevents the passage of UV rays through the textile. A sufficient level of wearing comfort must be considered [4,11]. In addition, an important factor is the color of fibers, dyeing, and printing material [4,12,13,14], where darker colored materials offer significantly better protection [8,13]. However, it is true that dark (e.g., black) colored textiles absorb UV radiation strongly, which they then convert into heat, making us warmer in such clothing. Water content, water vapor, sweat, the presence of various additives [7,13,15,16] (absorbers, repellents), etc. and changes that occur during wear [2,17], all this affects the changes in the protection of textiles from UV radiation.

Many studies [1,4,11,12,18,19,20,21] have shown that the aforementioned textile parameters have different effects on the protective properties of textiles, with some parameters having a greater influence on protection and others having less. For the development of a method to predict the protection against UV rays at the stage of the design and construction of fabrics (before production), it is necessary to know all the parameters that affect the UV protection of the fabric. Roughly speaking, adequate protection against UV radiation is most easily and quickly achieved by an appropriate textile construction, which must therefore have a sufficient cover factor, and/or using protective agents that allow a high reflection (R) or absorption (A) of UV radiation.

The aim of the research was to develop a fast, universal, and efficient method for predicting the UV protective properties of fabrics at the planning and design stage. The method is based on the determination of the parameters of UV radiation transition (transmission, reflection, and absorption) through textiles, more precisely through thread/yarn systems. For this purpose, it was necessary to extend the existing cover factor theory by considering that the transition through parts of the surface covered by one and two yarns is different. The method is based on the extended cover factor theory and the basic preparation of fabric samples or threads systems with previously known construction parameters of samples.

The specific problem and main result of this work is the determination of the K_R_, K_T_, and K_A_ for different row materials and types of yarns. That allows us to establish a simplified mathematical model for predicting the UV protective properties of woven fabrics based on the set of special fabrics with the possibility of predicting parameters of protection from UV radiation in advance. In this case, we have introduced the method of preparing the simulation of fabrics of any yarns and in this way simple, quick, and cheap determination of K_R_, K_T_, and K_A_.

The theoretical calculations give us the relations between almost all parameters of UV penetration through fabrics, but in practice, we do not get unified results from all invented relations. They are all very close to each other, but we want to find the best procedure to obtain the results in the simplest and most accurate way, also in regard to the number of samples and the time for their preparation.

## 2. Theoretical Part

### 2.1. Extended Woven Fabric Cover Factor Theory

**Figure 1:**
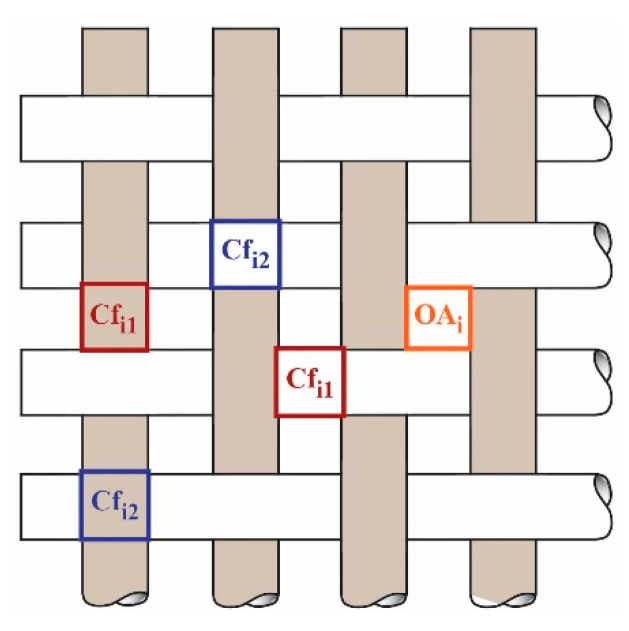
Scheme of woven fabric in plane weave, where there are shown areas without yarns (open area (OA) = $\sum^{\;}{\mathrm{OA}}_{i}$), units/areas of fabric surface covered by only one (Cf_1_ = $\sum^{\;}{\mathrm{Cf}}_{i1}$ ) and two yarn (Cf_2_ = $\sum^{\;}{\mathrm{Cf}}_{i2}$ ) where i is the number of individual areas in a unit area of fabric.

Cover factor (i.e., the degree of fabric fullness (Cf)) is a proportion of the fabric area covered by warp and weft yarns. Classical woven fabric cover factor theory is defined as the ratio between the projected area of the two elementary yarn systems in the fabric area unit [22,23]. It means that in practice, cover factor is calculated independently for warp and weft yarn by the proportion of fabric area covered by those yarn [22]. Theoretically, it can be calculated from the known yarn density and the theoretical yarn diameter. Due to the insufficient description of the yarn-covered area by the classical existing cover factor theory, the need for an extension of the cover factor theory became apparent. This considers the penetration of UV radiation and/or light through the mesh structure of the fabric as equivalent, although the surface/area coverage of biaxial fabrics (single-layer woven fabrics) with one system of warp and weft yarns consists of surface units covered by only one yarn (warp or weft) and the corresponding number of surface units covered by two yarns (warp and weft) simultaneously (Figure 1) [24].

Generally, the theory of woven fabrics construction is defining a cover factor as a fraction of surface covered by yarns which is equal to quotient of area covered by yarns and total area of web [22] expressed by Equation (1). That way, it takes the values from 0 to 1, and if it is multiplied by 100, it is expressed in %.

(1) Cf = area covered by yarns/total area of web

Calculation of the cover factor consists of the calculation of warp and weft cover factor as Cf_wa_ = D_wa_ × d_wa_ and Cf_we_ = D_we_ × d_we_ where D_wa_ and D_we_ are densities of warp and weft and d_wa_ and d_we_ are the diameter of warp and weft yarns.

The cover factor of a single-layer fabric is expressed by the cover factor of warp and weft yarns. It transforms Equation (1) to the form of Equation (2):

(2) $$\mathrm{Cf}\;={\mathrm{Cf}}_{\mathrm{wa}}+{\mathrm{Cf}}_{\mathrm{we}}-{\mathrm{Cf}}_{\mathrm{wa}}\times {\mathrm{Cf}}_{\mathrm{we}}.$$

For the purpose of investigation, we had to modify and expend woven fabric cover factor theory to the next form. The fabric cover factor is the sum of areas covered by one yarn—Cf_1_ and two yarns—Cf_2_ expressed with Equation (3):

(3) $$\mathrm{Cf}\;={\;\mathrm{Cf}}_{1}+{\mathrm{Cf}}_{2}$$

The portion of fabric covered by two yarns is calculated as shown in Equation (4):

(4) $${\mathrm{Cf}}_{2}\;={\;\mathrm{Cf}}_{\mathrm{wa}}\times {\mathrm{Cf}}_{\mathrm{we}}$$

The portion of yarns covered by one yarn as shown in Equation (5):

(5) $${\mathrm{Cf}}_{1}\;=\;\mathrm{Cf}-{\mathrm{Cf}}_{2}$$

This extended cover factor theory allows for a completely new theoretical approach to the treatment of the individual portions of the cover factor in the fabric structure for a certain group of fabrics—e.g., square fabrics or fabrics made of equal warp and weft yarns/threads and densities, which are used in practice for fine filtration or printing screens. For that, Equation (2) transforms in Equation (6):

(6) $$\mathrm{Cf}\;=\;2\times {\mathrm{Cf}}_{\mathrm{wa},\mathrm{we}}-{\mathrm{Cf}}_{\mathrm{wa},\mathrm{we}}{}^{2}$$

**Figure 2:**
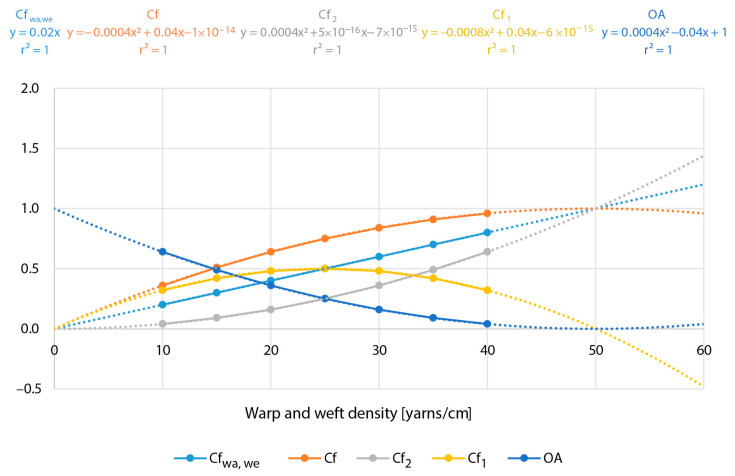
Cf_wa,we_, Cf, Cf_1_, Cf_2_ and OA, depending on D with a linear line of Cf_wa,we_ and other curves (second-order polynomial functions) and the corresponding coefficient of determination.

To explain it graphically, we chose fabrics with an ideal theoretical diameter up to (200 µm) and different densities of warp and weft (10, 15, 20, 25, 30, 35, and 40 yarns/cm). Their theoretically calculated values of the cover factors—warp and weft cover factor (Cf_wa_,_we_), fabric cover factor (Cf), and cover factor, which refers to the proportion of the area of the fabric covered by only one (Cf_1_) and two (Cf_2_) yarns and the open area (OA) of fabric—are given in Table 1, and the graph of the curves as a function of the different density is shown in Figure 2.

The values shown in Table 1 belong to the real data or yarn parameters and densities from which real fabrics can be made in almost any weave. To produce fabrics, it is possible to use lower densities, but their usability would be questionable, or higher densities than those listed, but in this case, it would be difficult, if even not impossible, to produce them, since in the case of plain weave at densities of 25, 30, and 35 yarns/cm, there may be heavy force to the weft beat-up and excessive yarn deformation. Therefore, in Figure 2, the values of the curves shown were interpolated (dotted part of the curves) (for a density equal to 0 yarns/cm to obtain the value zero, and for a density of additional 20 yarns/cm, to show the prediction of higher densities and all cover factors and open area). Figure 2 shows that only the cover factor of the Cf_wa,we_ as a function/dependent of density is a linear line, and all other curves are second-degree polynomials. The straight line intersects the second-degree parabolas for the Cf and Cf_2_ at a single point corresponding to the maximum density of ideal yarns with a diameter of 200 μm, i.e., 50 yarns/cm. At this point, the parabola of the curve coefficient Cf reaches the maximum value and allows calculating the maximum density also from the first derivative of an equation of the Cf curve written in Figure 2 (y = −0.0004x^2^ + 0.04x − 1 × 10^−14^; x = 50). The opposite is true for the OA curve as a function/dependent on the yarn density. Curve—the cover factor related to the Cf_2_ as a function/dependent on density, parabolically increasing. Curve—cover factor related to the Cf_1_, as a function/dependent on density, theoretically decreasing with increasing density up to the point where Cf_2_ equals 1—at this point Cf_1_ = 0. From the equation curve—cover factor related to the Cf_1_, as a function/dependent on density shown in Figure 2, at the value Cf_1_ = y = 0, we calculate the ideal maximum density (y = −0.0008x^2^ + 0.04x − 6 × 10^−15^; x = 50 yarns/cm) or half of the maximum density, by considering the first derivative of the mentioned curve equation (where we obtain x = 25 yarns/cm). 

### 2.2. Introduction of Simplified Mathematical Model for Predicting UV Properties of Woven Fabrics

In a previous work [11,24], we evaluated a penetration of UV rays through single layer monofilament PET fabrics trying to find out how much is reflected, absorbed, and transmitted by one yarn and how much from the two yarns in the fabric structure. We calculated quotient as shown in Equations (7)–(9) for every sample.

(7) $$K_{\mathrm{Tm}}=\left(T-\mathrm{OA}\right)/\mathrm{Cf}\;$$

(8) $$K_{\mathrm{Rm}}=R/\mathrm{Cf}$$

(9) $$K_{\mathrm{Am}}=A/\mathrm{Cf}$$

where K_Tm_, K_Rm_, and K_Am_ are the coefficient of transmission, reflection, and absorption of material (yarns from which fabrics is made); T and R are the measured values of transmission (T) and reflection (R) and calculated absorption (A) as a difference from 100; Cf is the cover factor of woven fabric. 

The physical meaning of quotient is how much the fabric/material with a certain structure will reflect, absorb, and transmit UV rays in case there is no open area in the sample or when Cf is equal to 1. In such a supposed case, the quotients should be equal to measured values. In the simplified mathematical model for predicting UV properties of woven fabrics, we took the values for K_1R_, K_1T_, and K_1A_ as constant for some material and assume that the transmission of the fabric area covered with two yarns (K_2T_) is approximately equal to the power function of the coefficient of the UV transmission of the fabric area covered with one yarn (K_1T_^2^), and that the coefficient of the UV absorption of the fabric area covered with two yarns (K_2A_) is approximately equal to the square root of the coefficient of the UV absorption of the fabric area covered with one yarn (√K_1A_).

**Figure 3:**
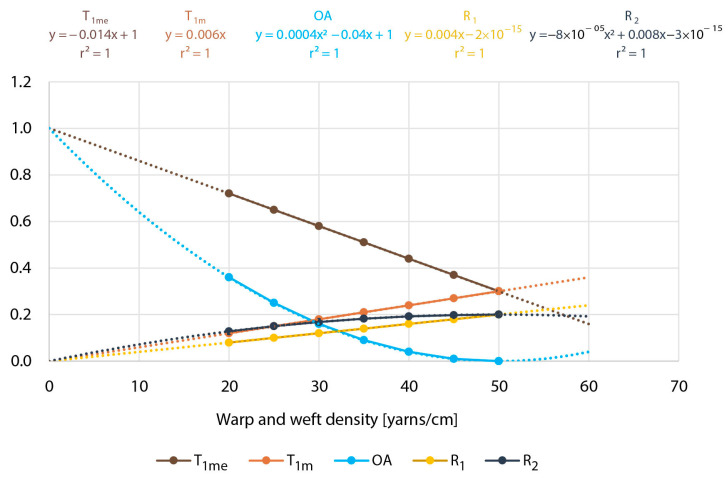
Curves of reflection from one-layer (R_1_), double-layer (R_2_) samples, T_1m_, T_1me_, and OA of double-layer samples.

The reflection coefficient from covering with one and two yarns remains the same K_1R_ = K_2R_ = K_R_. There is no absolute/internal trough; all values are approximately as explained, and therefore, we call the model simplified. However, we hope that the real values are so close to real that the mistake in the prediction is not more than a few percent, which is good enough for the purpose of predicting the UV properties. To confirm this, we will use a kind of simulation as we previously did regarding cover factor. We will suppose that the cover factor elements will stay the same as in Table 1 and Figure 2 and take the next values for K_1R_ = 0.2; K_1T_ = 0.3; and K_1A_ = 0.5. The results of simulation are shown in Table 2 and Figure 3 according to Equations (10)–(17):

(10) $$T_{1\mathrm{me}}\;=\;1-{\mathrm{Cf}}_{\mathrm{wa},\mathrm{we}}+{\mathrm{Cf}}_{\mathrm{wa},\mathrm{we}}\times K_{1T}$$

(11) $$T_{2\mathrm{me}}\;=\;\mathrm{OA}+{\mathrm{Cf}}_{1}\times K_{1T}+{\mathrm{Cf}}_{2}\times K_{1T}^{2}$$

(12) $$T_{1m}\;={\;\mathrm{Cf}}_{\mathrm{wa},\mathrm{we}}\times K_{1T}$$

(13) $$T_{2m}\;={\;\mathrm{Cf}}_{1}\times K_{1T}+{\mathrm{Cf}}_{2}\times K_{1T}^{2}$$

(14) $$A_{1}\;={\;\mathrm{Cf}}_{\mathrm{wa},\mathrm{we}}\times K_{1A}$$

(15) $$A_{2}\;={\;\mathrm{Cf}}_{1}\times K_{1A}+{\mathrm{Cf}}_{2}\times {\sqrt{K}}_{1A}$$

(16) $$R_{1}\;={\;\mathrm{Cf}}_{\mathrm{wa},\mathrm{we}}\times K_{R}$$

(17) $$R_{2}\;=\;\mathrm{Cf}\times K_{R}$$

From Table 2, it is evident that the summa of T_1_, R_1_, A_1_, and open area for single-layer samples (1 − C_wa,we_) is always equal to 1, and for double-layer samples, it is more than 0.99. In the last row, it is seen that the values for one-layer samples are identical as were supposed and that the transmission trough double-layer samples correspond to the squared function of K_1T_ and square root function of K_1A_.

Usually, in the real samples, we can easily determine only the density of yarns and not the real deformed diameter of yarns as well as K_R_, K_T_, and K_A_. What is measured is open area—optically, and transmission and reflection spectrophotometrically. The curves presented in Figure 3 gave us the possibility of easily detecting the missing parameters necessary for calculating the cover factor elements and predicting UV protective properties.

Figure 3 shows that there are several possibilities to identify the diameter of yarns and also to verify it by double or triple checking. We will list some of them:

- Optically: from the single-layer samples, we can get linear curve of C_wa,we_. At the point where the linear curve becomes 1, we can get the maximum density and also the proper diameter of yarn (Equation (18)). This is also possible following the double-layer curve (Equation (19)) [25]:
  (18) $$d\;=\;\frac{1}{D_{max}}$$
  (19) $$d_{\mathrm{wa},\;\mathrm{we}}\;=\;\frac{\left(1-\sqrt{O_{p}}\right)}{D_{\mathrm{wa},\;\mathrm{we}}}$$
- Determining the maximum of reflection curve from the double-layer systems of yarns—R_2_, that way, calculating the maximal density, diameter of yarns, and value of K_R_. (R_2_ = −0.00008x^2^ + 0.008x, from the first derivation of the equation of the function curve R_2_′ = −0.00016x + 0.008 = 0, we calculate x (maximal density of yarns) x = 50 and K_R_ = 0.2).
- Supposing the reflection from single-layer and double-layer fabrics at cover factor 1 will be equal, we can verify the previous mentioned findings using the same values when curves for R_1_ and R_2_ cross each other (−0.00008x^2^ + 0.008x = 0.004x, where we get x = 50 in K_R_ = 0.2).
- If we determine T_1m_ by subtraction T_1me_ for OA of single-layer samples, we can see that the linear curves for T_1me_ and T_1m_ cross each other in the point that corresponds to maximal density and to the value of K_R_ at the same time (−0.14x + 1 = 0.006x, follows x = 50 and K_T1_ = 0.3).

Of course, all statements above are valid for square fabrics (fabrics from the same yarns and same densities in warp and weft construction). The pallet of such structured fabrics practically does not exist, so we developed a method for creating such type of samples allowing presented findings and calculations.

### 2.3. Development of Method for Creating Samples Proper for Processing

To produce the fabric samples, we used a Minifaber laboratory loom (Minifaber, Italy), which has a relatively regular yarns arrangement per unit length—uniform density of the weft.

**Figure 4:**
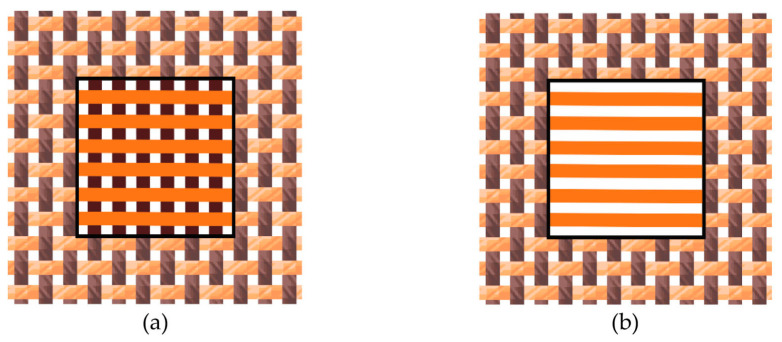
Preparation of thread system samples for measurements: (**a**) woven fabrics with floating warp and weft yarns; (**b**) Removing the warp and display measuring surface of thread distribution—thread system sample.

The yarns whose UV radiation parameters have to be measured were woven only in the direction of the weft by allowing their flotation (non-interlacing) in the size of 2 × 2 cm^2^ (selected part on Figure 4a). We weave fabric samples with different densities of the weft and with a constant density of the warp (20 yarns/cm). To achieve uniform thread density and tension in the measurement process, we embedded the samples in the frame. In the thread flotation areas, the warp yarns were removed from the fabric samples (Figure 4b). We used the samples prepared to measure UV transmittance and reflectance.

**Figure 5:**
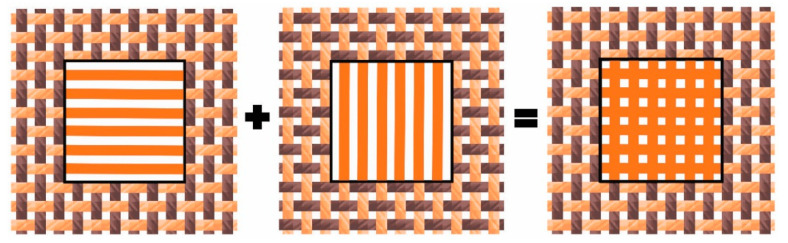
Schematic representation of the preparation of double-layer samples.

We measured the UV radiation parameters on single-and double-layer yarn systems. For double-layer samples, we laid two single layers crosswise (at an angle of 90°) with the approximation of fabric simulation from the same yarns and with the same density (Figure 5). The thread in the vertical and horizontal directions was the same. We wanted to simulate the fabric without interlacing and thus avoid the conditional deformation that is realistically reflected in the fabric.

## 3. Materials and Methods

To support the model presented and confirm the theory, we used two different series of samples and material. The first set of samples was made from red-colored CO yarns and was deliberately chosen to check how high can be the effect of UV protection. Cotton is the ideal material for summer cloths but allows high transmission of UV radiation when undyed. Dyed yarns extensively increase the absorption of UV radiation on account of diminishing its transmission [12]. 

The second set of samples from the PET rotor yarn was also chosen deliberately to check the K_R_, K_T_, and K_A_ determined previously in one of our research studies for monofilament PET fabrics [11,24]. The necessary data for chosen materials and methods are presented below.

### 3.1. Materials

For the experimental work, we prepared samples with the same construction parameters in both (warp and weft) directions, so-called square fabrics. We used a Minifaber laboratory loom (Minifaber, Italy) to produce the samples.

**Figure 6:**
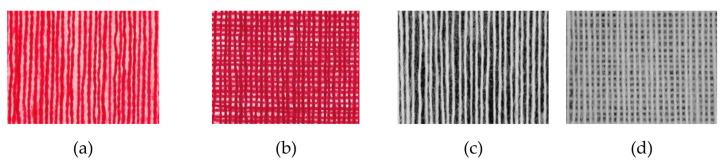
Single-layered (**a**,**c**) double-layered yarn systems (**b**,**d**) from colored cotton (CO) yarns (**a**,**b**) and undyed polyester (PET) yarns (**c**,**d**) in 10× magnification.

For the first series of weaved samples, we used dyed CO ring yarns, fineness 2 × 8 tex in four densities (20, 25, 30, and 35 yarns/cm) as single- (Figure 6a) and double- (Figure 6b) layer yarn systems. For the second series of weaved samples, we used raw, undyed PET rotor yarn, fineness 34 tex, in three densities (6, 10, 14, and 18 yarns/cm) as single- (Figure 6c) and double- (Figure 6d) layer yarn systems. A method for the preparation of single- or double-layer samples with uniform density was developed and used (Section 2.3) [26,27].

### 3.2. Methods

The transmission and reflection of the selected samples was measured with Lambda 800, UV/VIS Spectrophotometer, PELA-1000 (PerkinElmer Inc., Waltham, MA, USA). Measurements were made an “in vitro” method in accordance with the SIST EN 13758-1:2002 standard, in 2-nm steps and in the range of 700–200 nm. We made five measurements on each sample (vertically, horizontally, and at an angle of 45°).

## 4. Results and Discussion

In the following results, we do not expect such ideal matches and values as we have obtained in theory, for we must take into account the unevenness of the yarn, their harness, and possible mistakes in the preparation of the samples. Special attention must be paid to the accurate calculation of the real densities, as they have an extreme influence on the calculated results. A single deviation can affect the entire calculation.

Considering the possibilities of calculating the maximum density and cover factor mentioned in Section 2.2 and the small differences resulting from the above-mentioned reasons, different values of the maximum density calculated by spectrophotometric and optical methods are to be expected. This raises the question of what data can be used for the calculations. Since the primary object of research was the protection from UV radiation and this is directly related to the UV transmittance of the fabrics, we choose directly measured spectrophotometric results for the determination of the maximum density and proper diameter of the yarns.

The results of declared (D_d_) and measured (D_me_) density and measured values of the T and R for single- and double-layered CO samples are presented in Table 3.

**Figure 7:**
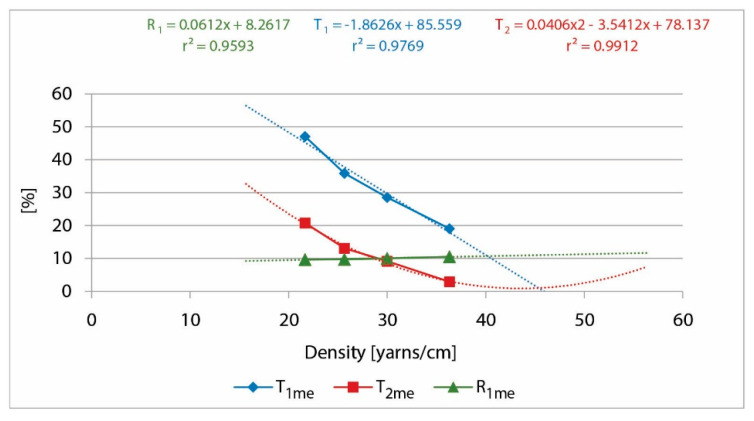
T and R of UV radiation of single-layer and double-layer CO samples, depending on the thread density, with the corresponding linear, polynomial equations, and the coefficient of determination.

From the measured values of T_1_, T_2_, and R_1_ presented in Table 3, we created a graphical representation (Figure 7) and got the equation describing them in dependence of density. The maximum density (D_max_) was calculated from derivation of the T_2_ curve, because at that point, the OA through samples supposed to be a zero, and the transmission should be minimal. At the same time, the obtained results are based on the original measurement. Table 4 presents values for K_2T_ ≈ K_1T_^2^ and K_2A_ ≈ √K_1A_ following the theory presented in Section 2.2. These values, D_max_ and d_min_, were taken for the further calculation of the cover factor parameters presented in Table 5. From the linear equation T_1_ and R_1_, we calculated K_1T_ and K_1R_ at the point of determined maximum density and K_1A_ as different from 1 (Table 6).

Table 5 and Table 6 show the calculation values of T_2_, R_2_, and A_2_ (Equations (11), (15) and (17)) according to the theory. Table 6 presents calculated values compared with measured values. It also presents the absolute differences among calculated and measured values as well as correlation among them. It is obvious that the level of mistakes does not exceed 6.7% at the lowest used density. 

The obtained results clearly confirm the known fact that colored cotton yarns can be used for permanent and successful protection against UV radiation if the woven fabric construction is carefully planned. For example, if the weave is sateen, fabric construction can have a maximum weft density and because of the high coefficient of absorption can offer practically excellent protection against UV radiation [12].

**Figure 8:**
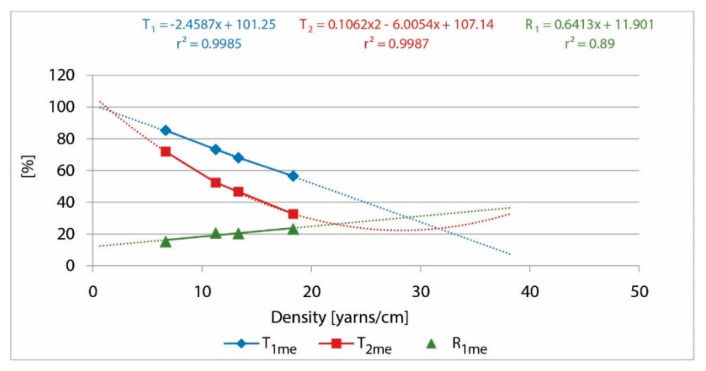
T and R of UV radiation of single-layer and double-layer PET samples, depending on the yarn density, with the corresponding linear, polynomial equations, and the coefficient of determination.

For the second set of samples from PET yarns, we used the same methodology as for the first set of samples from CO yarns. The results are presented in Table 7, Table 8, Table 9 and Table 10 and in Figure 8.

The presented research shows very high correlations of 0.98 and differences not higher than 5% between the calculated and measured values, which confirms the theoretical assumption and confirms the established mathematical model for the prediction of the UV protective properties of fabrics.

The identified coefficient of transmission for rotor PET yarn was 0.315, which was very close to the monofilament PET yarn (0.3–0.35) [11,24], confirming that the row material has a crucial role in the parameters of UV protection.

## 5. Conclusions

In the paper, an extension of the cover factor woven fabric theory was presented, which is necessary for understanding the mechanism of penetration of UV light through the yarn covered part of the mesh structure of fabrics. Regarding the penetration of UV light, the previous cover factor theory did not distinguish between the fabric areas covered by one and two yarns, which is why there was no existing model for predicting the transmission and thus the ultraviolet protection factor (UPF). The extension of the theory provides the theoretical relationships between the existing and newly introduced parameters of the cover factor (Cf, OA, Cf_1_, Cf_2_) and fabrics constructional parameters (d and D)). The theory was applied to the woven fabrics made from the same yarns and with the same warp and weft densities but can be easily extended to other types of woven fabrics.

A simplified theoretical model for the prediction of UV protection properties was presented, which offers the possibility to plan for the phase of fabric design stage. The model was called simplified because not all elements were exact; however, the calculations were very simple, and the results were good enough to serve the purpose. In addition to the elements of the extended cover factor theory, the data for the UV reflection, transmission, and absorption of the yarns (K_R_, K_T_, K_A_) were included in the model. Such data do usually not exist in the literature. The paper also presents the procedure of preparation of proper single- and double-layer samples. The samples represent a simulation of woven fabrics without interlacing of yarns (thus avoiding the deformation of yarns), which are suitable for fast and cheap determination of the missing data from a very low quantity of any row material and yarn type.

The experimental part performed on red dyed CO yarns confirmed the fact that intensely dyed yarns absorb most of the UV light (coefficient K_1A_ = 0.84), have small reflection (K_1R_ = 0.12) and very low transmission (K_1T_ = 0.04), which makes them almost perfect for constructing woven fabrics that can provide at least good, very good, and permanent UV protection. The results of the experiment on the second set of samples confirmed the assumption that coefficient of transmission of PET yarns is about 0.3 to 0.35, which was found in one of our previous research for the PET monofilament.

The obtained results of the experimental part justify both theories (cover factor and simplified model). It must be said that the obtained results practically give the maximal transmission (minimal UPF). Due to the interlacing in the real fabrics, the deformation of the yarns at the interlacing points and the resulting reduction of the open area, the transmission should be lower and the UPF should be higher.

## Figures and Tables

**Table 1:** Calculated Cf_wa,we,_ Cf, Cf_1_, Cf_2_, and open area (OA) depending on the given ideal yarn diameter (d) and different densities of warp and weft (D).

d [µm]	D [yarns/cm]	Cf_wa,we_	Cf	Cf_1_	Cf_2_	OA
200	10	0.20	0.36	0.32	0.04	0.64
	15	0.30	0.51	0.42	0.09	0.49
	20	0.40	0.64	0.48	0.16	0.30
	25	0.50	0.75	0.50	0.25	0.25
	30	0.60	0.84	0.48	0.30	0.16
	35	0.70	0.91	0.42	0.49	0.09
	40	0.80	0.96	0.32	0.64	0.25

**Table 2:** Transmission (T_me_—measured, T_m_—material), absorption and reflection values for simulated single- and double-layer samples calculated by the simplified model for the prediction of woven fabrics UV properties.

D_wa,we_ [yarns/cm]	T_1me_	T_2me_	T_1m_	T_2m_	A_1_	A_2_	R_1_	R_2_
20	0.72	0.5184	0.12	0.1584	0.2	0.3531	0.08	0.128
25	0.65	0.4225	0.15	0.1725	0.25	0.4268	0.1	0.15
30	0.58	0.3364	0.18	0.1764	0.3	0.4946	0.12	0.168
35	0.51	0.2601	0.21	0.1701	0.35	0.5565	0.14	0.182
40	0.44	0.1936	0.24	0.1536	0.4	0.6126	0.16	0.192
45	0.37	0.1369	0.27	0.1269	0.45	0.6628	0.18	0.198
50	0.30	0.09	0.30	0.09	0.50	0.7071	0.20	0.200

**Table 3:** D_d_ and D_me_ values of the transmission T (%) and reflection R (%), for single- (T_1_, R_1_) and double- (T_2_, R_2_) layered CO samples.

D_d_ [yarns/cm]	D_me_ [yarns/cm]	T_1_ [%]	T_2_ [%]	R_1_ [%]	R_2_ [%]
20	21.67	47.06	20.8	9.67	10.46
25	25.67	35.87	13.08	9.74	11.00
30	30.00	28.52	9.16	10.06	10.99
35	36.33	19.06	2.89	10.53	11.03

**Table 4:** Calculated values of K_T_, K_R_, and K_A_ for CO samples.

K_1T_	K_2T_	K_1R_ = K_2R_	K_1A_	K_2A_
0.04329	0.00187	0.10931	0.84740	0.92054

**Table 5:** D_max_ and d_min_, calculated values of Cf, Cf_wa_, Cf_we_, Cf_1_, Cf_2_, and OA for CO samples.

D_max_ [yarns/cm]	d_min_ [µm] _(Equation (18))_	Cf_wa,we_ [%]	Cf [%]	Cf_1_ [%]	Cf_2_ [%]	OA [%]	OA_1_ [%]
43.61	0.02293	49.69	74.69	50.00	24.69	25.31	50.31
		58.86	83.08	48.43	34.65	16.92	41.14
		68.79	90.26	42.94	47.32	9.74	31.21
		83.30	97.21	27.82	69.40	2.79	16.70

**Table 6:** Calculated values (_c_) of UV parameters double- (T_2_, R_2_, A_2_) layered CO samples and the absolute values of their differences compared with measurement (_me_) values and the conformation of their connections with the coefficient of correlation.

D_d_ [yarns/cm]	T_2_ _(Equation (11))_	R_2_ _(Equation (17))_	A_2_ _(Equation (15))_	Δ|T_2c_ − T_2me_|	Δ|R_2c_ − R_2me_|	Δ|A_2c_ − A_2me_|
21.67	0.27522	0.08164	0.65097	0.06722	0.02296	0.03643
25.67	0.19085	0.09081	0.72933	0.06005	0.01919	0.02987
30.00	0.11688	0.09866	0.79947	0.02528	0.01124	0.00097
36.33	0.04121	0.10626	0.87454	0.01231	0.00404	0.01374
r (_c:me_)	0.995	0.827	0.996			

**Table 7:** D_d_ and D_me_, values of the T (%) and R (%), for single- (T_1_, R_1_) and double- (T_2_, R_2_) layered PET samples.

D_d_ [yarns/cm]	D_me_ [yarns/cm]	T_1_ [%]	T_2_ [%]	R_1_ [%]	R_2_ [%]
6	6.67	85.26	71.94	15.28	16.12
10	11.25	73.64	52.30	20.68	22.57
14	13.33	68.03	46.65	20.33	24.47
18	18.33	56.58	31.55	23.11	28.40

**Table 8:** Calculated values of K_T_, K_R_, and K_A_ for PET samples.

K_1T_	K_2T_	K_1R_ = K_2R_	K_1A_	K_2A_
0.31564	0.09963	0.30056	0.38376	0.61948

**Table 9:** D_max_, d_min_, calculated values of Cf, Cf_wa_, Cf_we_, Cf_1_, Cf_2_, and OA for PET samples.

D_max_ [yarns/cm]	d_min_ [µm] _(Equation (18))_	Cf_wa,we_ [%]	Cf [%]	Cf_1_ [%]	Cf_2_ [%]	OA [%]	OA_1_ [%]
28.33	0.03530	23.28	41.14	35.72	5.42	58.45	76.45
		39.26	63.11	47.69	15.42	36.34	60.28
		46.52	71.40	49.76	21.64	28.03	52.94
		63.97	87.02	46.10	40.92	12.45	35.29

**Table 10:** Calculated values (_c_) of UV parameters double- (T_2_, R_2_, A_2_) layered PET samples and the absolute values of their differences compared with measurement (_me_) values and the conformation of their connections with the coefficient of correlation.

D_d_ [yarns/cm]	T_2_ _(Equation (11))_	R_2_ _(Equation (17))_	A_2_ _(Equation (15))_	Δ|T_2c_ − T_2me_|	Δ|R_2c_ − R_2me_|	Δ|A_2c_ − A_2me_|
6.67	0.70367	0.12488	0.12472	0.01573	0.03632	0.05205
11.25	0.53027	0.19133	0.25014	0.00727	0.03437	0.02710
13.33	0.45960	0.21632	0.31779	0.00690	0.02838	0.03527
18.33	0.31040	0.26313	0.50776	0.01580	0.02087	0.03667
r (_c:me_)	0.998	0.999	0.980			

## Data Availability

The data presented in this study are available on request from the corresponding author.

## References

[1] Postle R. Screening application of textile materials: An Australian perspective. Proceedings of the 4th ITC&DC.

[2] Urbas R., Sluga F., Bartenjev I. (2004). Influence of constructional parameters on UV protective efficiency of fabrics. Tekstilec.

[3] Downs C.A., Kramarsky-Winter E., Segal R., Fauth J., Knutson S., Bronstein O., Ciner F.R., Jeger R., Lichtenfeld Y., Woodley C.M. (2016). Toxicopathological effects of the sunscreen UV filter, oxybenzone (benzophenone-3), on coral planulae and cultured primary cells and its environmental contamination in Hawaii and the U.S. Virgin Islands. Arch. Environ. Contam. Toxicol..

[4] Dobnik Dubrovski P. (2010). Wowen Fabric and Ultraviolet Protection, Woven Fabric Engineering.

[5] Cox Crews P., Gwendolyn H. (2005). The ultraviolet protection factor of naturally-pigmented cotton. J. Cotton Sci..

[6] Wilson C.A., Bevin N.K., Laing R.M., Niven B.S. (2008). Solar protection—Effect of selected fabric and use characteristics on ultraviolet transmission. Text. Res. J..

[7] Pezelj E., Tomljenović A., Čunko R. (2004). Textiles for the protection against sun radiation. Tekstil.

[8] Dobnik-Dubrovski P., Golob D. (2009). Effects of woven fabric construction and color on ultraviolet protection. Text. Res. J..

[9] Majumdar A., Vijay K.K., Achintya K.M., Piyali H. (2012). Effect of weave, structural parameters and ultraviolet absorbers on vitro protection factor of bleached cotton woven fabrics. Photodermatol. Photoimmunol. Photomed..

[10] Kostajnšek K., Urbas R., Dimitrovski K. Modeling of UV protective properties of monofilament fabrics. Proceedings of the 8th ITC&DC.

[11] Dimitrovski K., Sluga F., Urbas R. (2010). Evaluation of the structure of monofilament PET woven fabrics and their protection properties. Text. Res. J..

[12] Gabrijelčič H., Urbas R., Sluga F., Dimitrovski K. (2009). Influence of fabric constructional parameters and thread colour on UV radiation protection. Fibres Text. East. Eur..

[13] Gorenšek M., Sluga F., Urbas R. (2007). Improving the ultraviolet protection factor of cotton fabric. AATCC Rev..

[14] Gies P. (2007). Photoprotection by clothing. Photodermatol. Photoimmunol. Photomed..

[15] Gorjanc M., Jazbec K., Mozetič M., Kert M. (2014). UV protective properties of cotton fabric treated with plasma, UV absorber and reactive dye. Fibers Polym..

[16] Hilfiker R., Kaufmann W., Reinert G., Schmdt E. (1996). Improving sun protection factors of fabrics by applying UV-absorbers. Text. Res. J..

[17] Kursun S., Ozcan G. (2010). An investigation of UV protection of swimwear fabrics. Text. Res. J..

[18] Dimitrovski K., Kostajnšek K. Evaluation of permeability properties of lightwait cotton fabrics with different construction. Proceedings of the 9th AUTEX 2009 World Textile Conference.

[19] Algaba I., Pepio M., Riva A. (2008). Correlation between the ultraviolet protection factor and the weight and thickness of undyed cellulosic woven fabrics. Fibres Text. East. Eur..

[20] Urbas R., Kostajnšek K., Dimitrovski K. (2011). Impact of structure and yarn colour on UV properties and air permeability of multilayer cotton woven fabrics. Text. Res. J..

[21] Kan C.W. (2014). A study on ultraviolet protection of 100% cotton knitted fabric: Effect of fabric parameters. Sci. World J..

[22] Peirce F.T. (1937). The geometry of cloth structure. J. Text. Inst. Trans..

[23] Dobnik-Dubrovski P. (2007). Konstrukcija Tekstilij.

[24] Kostajnšek K., Urbas R., Dimitrovski K. (2019). A New simplified model for predicting the UV-protective properties of monofilament PET fabrics. Autex Res. J..

[25] Kostajnšek K., Zupin Ž., Hladnik A., Dimitrovski K. (2021). Optical Assessment of Porosity Parameters in Transparent Woven Fabrics. Polymers.

[26] Kostajnšek K., Dimitrovski K. Fast effective method for predicting UV protective properties of woven fabrics. Proceedings of the 15th AUTEX 2015 World Textile Conference.

[27] Kostajnšek K., Logar D., Güngör B., Dimitrovski K. Determination of UV protective properties of yarns. Proceedings of the 16th AUTEX 2016 World Textile Conference.

